# Organ-Specific Expression of IL-1 Receptor Results in Severe Liver Injury in Type I Interferon Receptor Deficient Mice

**DOI:** 10.3389/fimmu.2019.01009

**Published:** 2019-05-09

**Authors:** Martina Anzaghe, Theresa Resch, Elea Schaser, Stefanie Kronhart, Clara Diez, Marc A. Niles, Eugenia Korotkova, Stefan Schülke, Sonja Wolfheimer, Dorothea Kreuz, Marion Wingerter, María Matilde Bartolomé Rodríguez, Zoe Waibler

**Affiliations:** ^1^Section 3/1 “Product Testing of Immunological Biomedicines”, Paul-Ehrlich-Institut, Langen, Germany; ^2^Vice President's Research Group 1 “Molecular Allergology”, Paul-Ehrlich-Institut, Langen, Germany; ^3^Section 3/3 “Morphology”, Paul-Ehrlich-Institut, Langen, Germany; ^4^Division of Veterinary Medicine, Paul-Ehrlich-Institut, Langen, Germany; ^5^Institute for Internal Medicine University of Freiburg, Freiburg, Germany

**Keywords:** anti-inflammatory, poly(I:C), immune pathology, dysregulation, cytokines

## Abstract

Upon treatment with polyinosinic:polycytidylic acid [poly(I:C)], an artificial double-stranded RNA, type I interferon receptor-deficient (IFNAR^−/−^) mice develop severe liver injury seen by enhanced alanine aminotransferase (ALT) activity in the serum that is not observed in their wildtype (WT) counterparts. Recently, we showed that liver injury is mediated by an imbalanced expression of interleukin (IL)-1β and its receptor antagonist (IL1-RA) in the absence of type I IFN. Here we show that despite comparable expression levels of IL-1β in livers and spleens, spleens of poly(I:C)-treated IFNAR^−/−^ mice show no signs of injury. *In vitro* analyses of hepatocytes and splenocytes revealed that poly(I:C) had no direct toxic effect on hepatocytes. Furthermore, expression levels of cytokines involved in other models for liver damage or protection such as interferon (IFN)-γ, transforming growth factor (TGF)-β, IL-6, IL-10, IL-17, and IL-22 were comparable for both organs in WT and IFNAR^−/−^ mice upon treatment. Moreover, flow cytometric analyses showed that the composition of different immune cells in livers and spleens were not altered upon injection of poly(I:C). Finally, we demonstrated that the receptor binding IL-1β, IL1R1, is specifically expressed in livers but not spleens of WT and IFNAR^−/−^ mice. Accordingly, mice double-deficient for IFNAR and IL1R1 developed no liver injury upon poly(I:C) treatment and showed ALT activities comparable to those of WT mice. Collectively, liver injury is mediated by the organ-specific expression of IL1R1 in the liver.

## Introduction

Type I interferons (IFN) are pro-inflammatory cytokines comprising 13–14 isoforms of IFN-α, IFN-β, and the lesser known IFN-ε, IFN-τ, IFN-κ, IFN-ω, IFN-δ, IFN-ζ, and IFN-v (1–6). Upon detection of pathogen-associated molecular patterns (PAMPs), IFNs can be induced by mainly two types of pattern recognition receptors: the toll-like receptor (TLR) family and the retinoic acid-inducible gene (RIG)-I-like receptors (RLR). PAMPs, such as double-stranded RNA, can be detected by TLR3 or the RLRs, RIG-I and melanoma differentiation-associated protein (MDA)5. Whereas, TLR3 is located within the endosome, RLRs are present in the cytoplasm.

All type I IFNs bind to one common type I IFN receptor (IFNAR) which consists of two chains, IFNAR1 and IFNAR2 (2). In a first wave, small amounts of type I IFNs, mainly IFN-α4 and IFN-β, are produced which bind to the IFNAR resulting in the production of large amounts of type I IFN (7, 8). Type I IFNs are able to induce the transcription of hundreds of type I IFN-stimulated genes (1, 2, 9) and therefore have pleiotropic effects in order to mediate resistance to viral replication and set up an overall anti-viral state.

Besides their pro-inflammatory actions, type I IFNs can also exert anti-inflammatory capacities. For example, IFN-β is used to treat patients with relapsing multiple sclerosis (10). In addition, type I IFNs suppress a wide range of pro-inflammatory and tissue-destructive activities of the potent pro-inflammatory cytokine and endogenous pyrogen interleukin (IL)-1 (11). The IL-1 family includes IL-1α and IL-1β that are produced as inactive pro-forms which are cleaved upon formation of the inflammasome into their bioactive forms (12). Both IL-1α and IL-1β bind to the IL-1 receptor type 1 (IL1R1). Once activated, IL1R1 associates with its co-receptor IL-1 receptor accessory protein (IL1RAcP) to create a transmembrane signaling complex that initiates IL-1-dependent pro-inflammatory immune responses. IL-1α and IL-1β are negatively regulated by their competitor IL-1 receptor antagonist (RA) that prevents IL-1-binding to IL1R1. In addition to IL-1RA, the decoy receptor IL1R2 can bind IL-1α or IL-1β (13, 14). Type I IFNs are able to prevent the secretion of IL-1β, both by hindering the production of pro-IL-1β and by blocking pro-IL-1β cleavage to mature IL-1β by preventing inflammasome activation (15). In a TLR9- and alcohol-induced model of liver injury, type I IFNs demonstrated a protective role via induction of IL-1RA and anti-inflammatory IL-10, respectively (16, 17).

Recently, we showed that poly(I:C) induces severe liver injury in IFNAR-deficient (IFNAR^−/−^) mice. The mechanism underlying poly(I:C)-induced liver damage was found to be an imbalanced expression of IL-1β and IL-1RA in the absence of type I IFN signaling (18). We demonstrated that myeloid-derived suppressor cells (MDSC) from the peritoneum infiltrate the liver, produce IL-1RA in a type I IFN-dependent manner, and thereby prevent liver injury. In contrast, IFNAR^−/−^ mice express high levels of IL-1β but not IL-1RA. Importantly, apart from the liver, all other organs showed no signs of injury (18). Here, we address the question how this strict organ specificity of IL-1β/IL-1RA action is mediated and how unaffected organs such as the spleen are protected. Understanding the mechanism(s) of organ-specific action of potentially harmful and/or protective cytokines will help to develop targeted treatment options with reduced side effects for patients with severe liver injury.

## Materials and Methods

### Mice and *in vivo* Applications

C57BL/6 wildtype (WT) mice were purchased from Harlan Winkelmann (Borchen, Germany). IFNAR^−/−^ mice (19) were backcrossed at least 20 times on the C57BL/6 background. IL1R1^−/−^ mice as described by Glaccum et al. (20) were purchased from Jackson Lab. To obtain IL1R1^−/−^IFNAR^−/−^ double-deficient mice, IL1R1^−/−^ mice were intercrossed with IFNAR^−/−^ mice. All mice were bred under specific pathogen free conditions at the Zentrale Tierhaltung of the Paul-Ehrlich-Institut. Correct gene knocks out were verified by PCR analysis for all genotypes used. Health monitoring results of sentinel mice for all knock-out mice showed no differences when compared to WT mice in our breeding. Fur structure, bearing, nutrition, and mating behavior of all knock-out mice were inconspicuous. Mouse experimental work was carried out using 8–12 week old mice in compliance with regulations of German animal welfare.

High molecular weight poly(I:C) (Invitrogen, France and AdipoGen Life Sciences, Germany) was dissolved in sterile PBS following manufacturer's instructions to a final concentration of 2 μg/μl. Mice were injected intraperitoneally (i.p.) with 15 μg poly(I:C) per gram body weight (BW) in a maximal volume of 200 μl. Recombinant human IL-1RA (Anakinra, kindly provided by Swedish Orphan Biovitrum) was diluted in PBS and i.p. injected 6 h before, simultaneously with, and 10 h after poly(I:C) treatment at a concentration of 75 μg/g BW in a maximal volume of 200 μl.

### Cell Isolation, Culture, and Stimulation

Isolation of hepatocytes was performed as described before (21, 22). For *in vitro* stimulations, 12-well plates were coated with collagen 24 h prior to cultivation. Hepatocytes were seeded at a density of 2 × 10^5^ cells/ml/12-well in William's E Medium supplemented with 10% FCS, 1% penicillin/streptavidin, 100 nM dexamethasone, and 1 μM insulin. After 4 h, cells were washed with PBS and 1 ml William's E medium supplemented with 100 mM dexamethason was added prior to stimulation.

For isolation of splenocytes, mice were euthanized by cervical dislocation and the abdomen was opened. The spleen was removed and splenocytes were obtained by dissection. The cells were resuspended in PBS and passed through a 70 μm nylon strainer. After centrifugation, red blood cells were lysed (RBC lysing buffer, Sigma-Aldrich, Munich, Germany) and cell counts were determined. Cells were seeded in 24-well plates at a density of 1 × 10^6^/ml in DMEM supplemented with 10% FCS, 1% penicilin/streptavidin (Gibco), and 1% GlutaMax (Gibco).

Liver perfusion was performed according to Fang et al. (23). In brief, animals were euthanized by CO_2_ inhalation and the abdomen was opened. Portal vein was cannulated using a 27G needle and 1 ml solution containing 10 mM EDTA and 100 U Heparin (Sigma) in PBS was injected. The right and the left renal veins were ligated with suture. The superior vena cava was also ligated with suture after the thorax was opened. Finally, the inferior vena cava was punctured with a 23G butterfly cannula. The liver was perfused with min. 10 ml 10 mM EDTA/PBS and the flow was collected with a syringe at the 23G butterfly cannula. The flow was centrifuged for 5 min at 400 g and the cell pellet was treated with BD Pharm lysing Solution (BD Biosciences) for red blood cell lysis. Upon red blood cell lysis, cells were washed and seeded in 96-well plates at a density of 2 × 10^5^/200 μl in DMEM supplemented with 10% fetal calf serum, 1% GlutaMax (Gibco), and 1% penicillin/streptavidin (Gibco). Hepatocytes and splenocytes were stimulated with 1, 10, and 100 μg/ml poly(I:C) for 24 h.

### Histology

For histological analysis, liver and spleen were fixed in 10% buffered formalin and embedded in paraffin. Sections were stained with hematoxylin and eosin (H&E) as described before (18) and examined by light microscopy.

### Flow Cytometry

Cells were stained for 30 min at 4°C with antibodies directed against CD4-PE (BD Pharmingen), CD8-FITC (MBL), CD19-FITC (Southern Biotech), and NK1.1-Alexa647 (Life Technologies). Cells were washed and analyzed with a LSRII flow cytometer (Becton Dickinson). Vitality was analyzed by staining with 7AAD (BD PharMingen) 5–10 min before FACS analyses. Analyses were performed using BD FACSDiva™ 8.0.1 and FlowJo^®^ 7.6.5.

### Quantification of Cytokine Production and ALT Activity

To determine serum cytokine levels and serum alanine aminotransferase (ALT) activity, peripheral blood was taken retro-orbitally upon anesthetization using Isofluran (CP-Pharma) and serum was prepared. ALT activity was determined using a commercially available kit (Hiss Diagnostics GmbH). Organs were ground in liquid nitrogen and lysed using RIPA-buffer. Supernatants were normalized to 5 μg/μl protein and stored at −80°C until cytokine measurement. Sixty-five percent donkey serum was added to the sample to prevent unspecific binding. Liver and spleen homogenates were tested for IFN-γ, TGF-β, IL-1β, IL-6, IL-10, IL-17, and IL-22 using commercially available Quantikine ELISA kits (all from R&D Systems). IL1R1 expression was analyzed using a commercially available ELISA (Aviva System Biology).

### Western Blot Analyses

Liver and spleen lysates of WT and IFNAR^−/−^ mice were generated by homogenization of organs in liquid nitrogen and subsequent lysis using RIPA-buffer. Samples were incubated for 5 min at 95°C in non-reducing loading buffer containing 10% SDS, and analyzed on a SDS-PAGE according to the method described by Laemmli (cross linker C = 5%, total bis/acrylamide = 15%) (24) followed by immunoblotting on nitrocellulose membranes (GE Healthcare, Freiburg, Germany). Detection of IL1R1 was performed using a rabbit polyclonal antibody against IL1R1 (1:1,000, Biozol, Germany) and a donkey-anti-rabbit-HRP secondary antibody (1:7,500, Amersham, Freiburg, Germany). Detection of actin was performed using mouse-anti-actin monoclonal antibody (1:1,000, Millipore, Germany) and a rabbit-anti-mouse-HRP secondary antibody (1:7,500, Thermo Fisher Scientific, Germany). After immunoblotting, samples were visualized with AceGlow chemiluminescence substrate (VWR, Darmstadt, Germany) by a CCD camera (Fusin-FX7 Spectra, Vilber Lourmat, Eberhardzell, Germany).

### Quantitative Real-Time PCR

Total RNA was prepared from liver and spleen using Trizol (Invitrogen)/Chloroform extraction. Samples were treated with DNaseI (Roche) for 15 min at 37°C. Absence of genomic DNA contamination was confirmed by standard PCR using a glyceraldehyde 3-phosphate dehydrogenase (GAPDH)-specific primer pair. Target- and reference-mRNA levels were examined by quantitative real time (qRT)-PCR using QuantiFast SYBR Green PCR kit (Qiagen). Primer pairs used were the following: GAPDH forward 5′-ACCACAGTCCATGCCATCAC-3′, GAPDH reverse 5′-TCCACCACCCTGT TGCTGTA-3′, pro-IL-1β forward 5′-TCTTTGAAGTTGACGGACCC-3′, pro-IL-1β reverse 5′- TGAGTGATACTGCCTGCCTG-3′, IL1R1 reverse 5′-CAG GTGGCAGAAATGCTAGA-3′. The expression levels of all target genes were normalized against GAPDH (ΔCt). Gene expression values were calculated based on the ΔΔCt method using the mean of the untreated control group as calibrator to which all other samples were compared. Relative quantities (RQ) were determined using the equation RQ = 2^−ΔΔ*Ct*^.

### Statistics

For statistical analyses the unpaired two tailed *t*-test was performed.

## Results

### IL-1β Expression in IFNAR^−/−^ Mice Upon Poly(I:C) Treatment Results in Liver Injury While the Spleen Remains Unaffected

**Figure 1:**
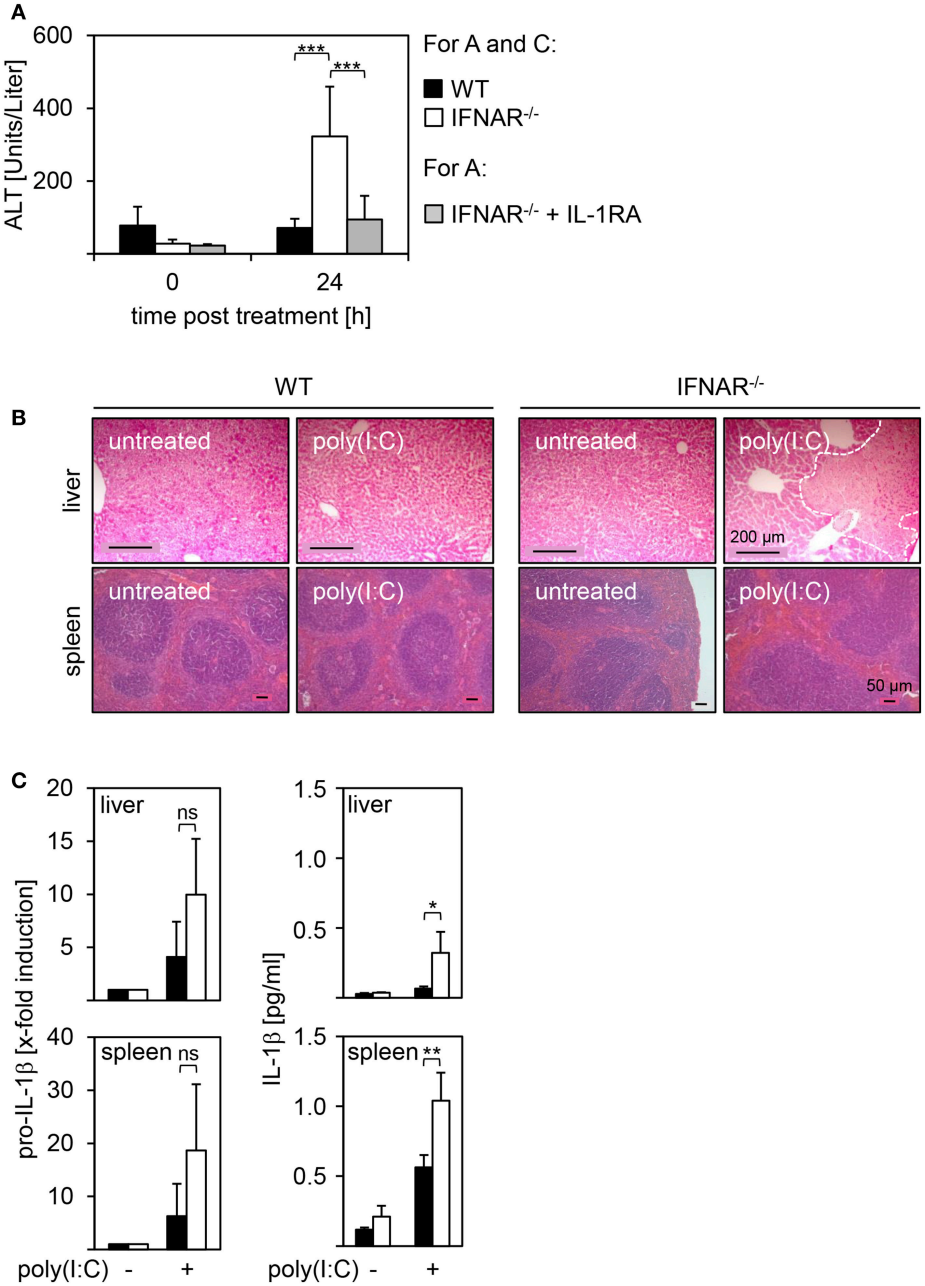
IL-1β expression in IFNAR^−/−^ mice upon poly(I:C) treatment results in liver injury while spleens remain unaffected. WT and IFNAR^−/−^ mice were injected with 15 μg/g BW poly(I:C). **(A)** Where indicated, IFNAR^−/−^ mice were treated with rIL-1RA. Serum was collected 24 hpi and ALT activity was measured (WT *n* = 7, IFNAR *n* = 11, IFNAR + rIL-1RA *n* = 8). **(B)** Organs were harvested 18 hpi. After 24 h of incubation in 10% formalin, organs were embedded in paraffin, 2 μm slices were prepared, and stained with H&E. Organs of untreated animals served as controls. Size standard is 200 μm for liver sections and 50 μm for spleen sections, respectively. Representative sections for 2–4 experiments are shown. The dotted line indicates necrotic areas. **(C)** Livers and spleens were harvested, homogenates or organ lysates were prepared, and analyzed for proIL-1β expression (4 hpi) by quantitative qRT-PCR (left side; *n* = 3) or IL-1β expression (10 hpi) by ELISA (right side; *n* = 3–4). Cytokine concentrations from liver lysates in **(C)** were already published (18). For **(A,C)**: Error bars indicate standard deviations. ns, not significant; ^*^ ≤ 0.05; ^**^ ≤ 0.01; ^***^ ≤ 0.001 (unpaired two-tailed *t*-test).

Recently, we showed that mice deficient for the IFNAR develop severe liver injury upon treatment with poly(I:C). In IFNAR^−/−^ mice, this is related to an imbalance in type I IFN-mediated IL-1β and IL-1RA expression, and injection of recombinant (r)IL-1RA prior to poly(I:C) rescues IFNAR^−/−^ mice from liver injury [(18) and Figure 1A]. Of note, other organs such as the spleen were unaffected by poly(I:C) treatment (18). H&E stained organ sections of poly(I:C)-treated WT and IFNAR^−/−^ mice confirmed that, in line with the enhanced ALT activity, the liver of poly(I:C)-treated IFNAR^−/−^ mice showed large necrotic areas while spleen architecture of both poly(I:C)-treated WT and IFNAR^−/−^ mice was indistinguishable from untreated controls (Figure 1B). The spleen represents an important secondary lymphoid organ harboring many immune cells. Hence, spleen was included in all further experiments as unaffected control organ. In order to investigate if dysregulation of IL-1β expression in absence of an intact type I IFN system is only present in the liver, livers and spleens of poly(I:C)-treated WT and IFNAR^−/−^ mice were analyzed for proIL-1β and IL-1β expression by qRT-PCR and ELISA. As given in Figure 1C left, poly(I:C) treatment resulted in an upregulation of proIL-1β mRNA in both liver and spleen, and was more pronounced in IFNAR^−/−^ mice when compared to their WT counterparts. In line with the qRT-PCR results, protein analyses indicated a significantly enhanced IL-1β expression in livers and spleens of poly(I:C)-treated IFNAR^−/−^ mice when compared to WT organs (Figure 1C right).

Collectively, poly(I:C) treatment of IFNAR^−/−^ mice induces high expression of IL-1β in both livers and spleens. Nevertheless, IL-1β expression results in severe injury of the liver only whereas spleens remain unaffected.

### Poly(I:C) Treatment of Hepatocytes and Splenocytes Does Not Induce Cytotoxicity

**Figure 2:**
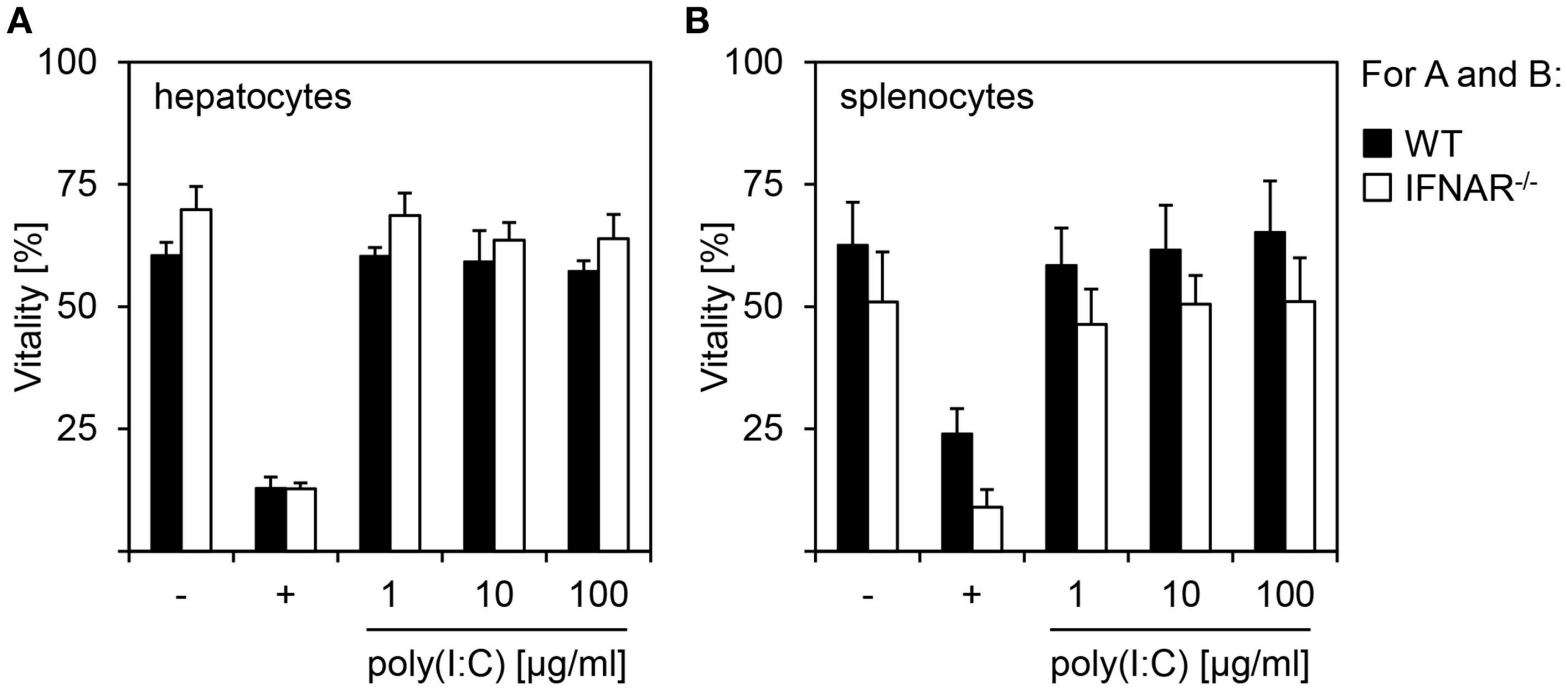
Poly(I:C) treatment of hepatocytes and splenocytes does not induce cytotoxicity. Hepatocytes **(A)** and splenocytes **(B)** were isolated from WT and IFNAR^−/−^ mice and stimulated *in vitro* with poly(I:C) at the indicated concentrations. Cells were left untreated (–) or treated with HCl (+) as controls. Vitality was determined by flow cytometry using 7AAD (*n* = 3–6, measured in three independent experiments).

In order to investigate if IL-1β-mediated organ-specific injury observed in poly(I:C)-treated IFNAR^−/−^ mice results from an increased toxicity in a specific cell or genotype, hepatocytes and splenocytes were isolated from WT and IFNAR^−/−^ mice. *In vitro* stimulations with poly(I:C) at different concentrations (1, 10, and 100 μg) for 24 h were performed and cells were analyzed by flow cytometry. As positive control for damage, cells were treated with HCl (+). As given in Figures 2A,B, poly(I:C) treatment did not reduce cell viability when compared to untreated cells. This was irrespective of the genotype of both hepatocytes and splenocytes. Hence, a direct toxic effect on (IFNAR^−/−^) hepatocytes can be excluded as the reason for the organ-specific damage upon poly(I:C) treatment.

### IFNAR^−/−^ Livers and Spleens Show a Comparable Cytokine Milieu Upon Poly(I:C) Treatment

**Figure 3:**
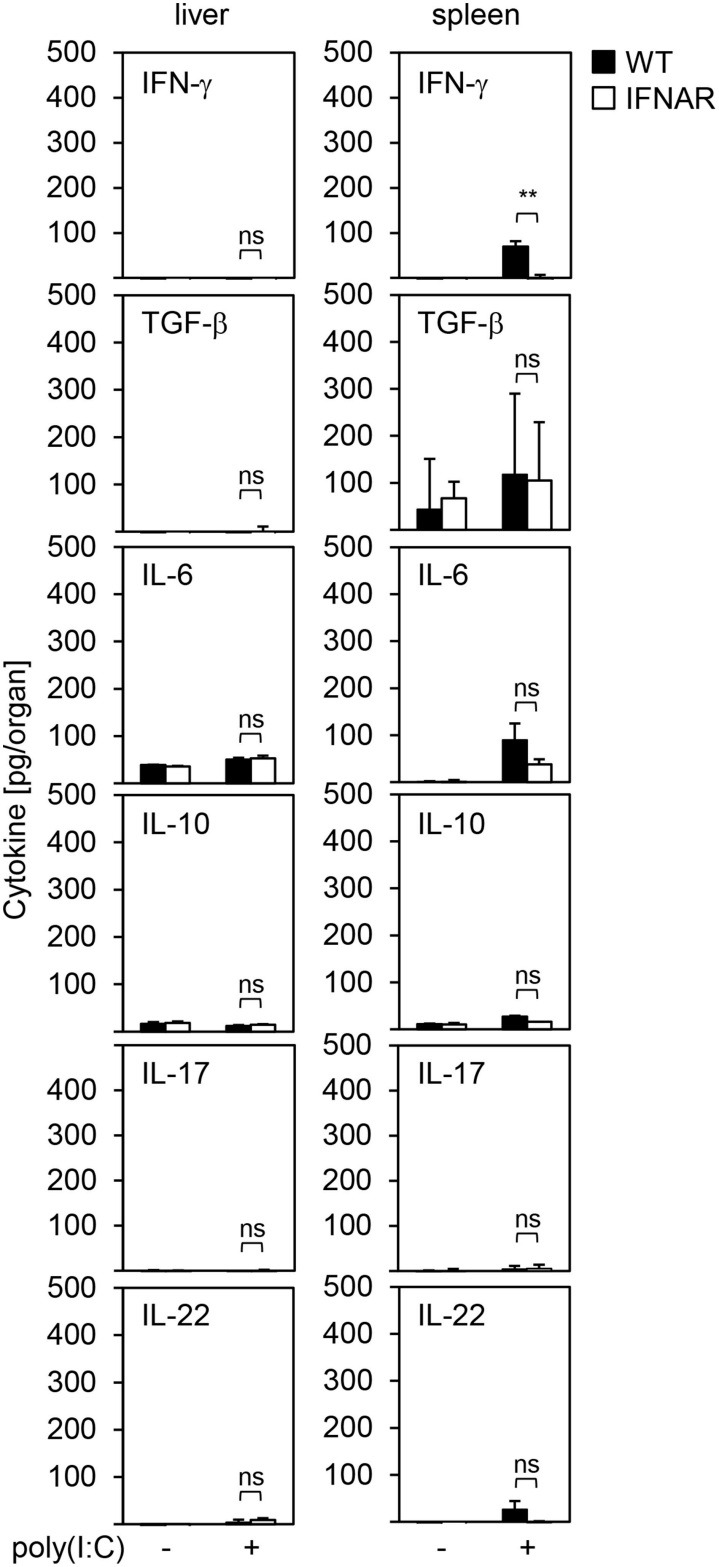
IFNAR^−/−^ livers and spleens show a comparable cytokine milieu upon poly(I:C) treatment. WT and IFNAR^−/−^ mice were injected with 15 μg/g BW poly(I:C). Livers and spleens were isolated 10 hpi and organ homogenates were analyzed for expression of the indicated cytokines by ELISA. Organs of untreated mice served as controls (*n* = 3–6). Error bars indicate standard deviations. ns, not significant; ^**^ ≤ 0.01 (unpaired two-tailed *t*-test).

It is well-accepted that besides IL-1β and IL-1RA other cytokines are associated with liver injury or protection as well. This has been reported for IFN-γ (25), transforming growth factor (TGF)-β (26, 27), IL-6 (28–30), IL-10 (31, 32), IL-17 (33), and IL-22 (34, 35). Hence, we analyzed the local expression of these cytokines in livers and spleens of untreated and poly(I:C)-treated WT and IFNAR^−/−^ mice. As shown in Figure 3, overall IFN-γ expression was very low in spleens and slightly upregulated in WT but not IFNAR^−/−^ spleens upon poly(I:C) treatment, whereas livers showed no IFN-γ expression. IL-6 was slightly upregulated upon poly(I:C) treatment in both WT and IFNAR^−/−^ spleens, while all other cytokines were not induced or reduced, respectively, upon treatment.

Of note, WT and IFNAR^−/−^ spleens showed some minor expression of TGF-β which was not observed in livers. Nevertheless, TGF-β expression levels were comparable between WT and IFNAR^−/−^ mice and similar to those observed in spleens of untreated control animals suggesting that the elevated expression of TGF-β rather represents a basal expression and is not involved in onset or protection from liver injury. Therefore, organ-specific damage of IFNAR^−/−^ mice is not mediated by differences in the cytokine milieus of liver and spleen upon poly(I:C) treatment.

### Organ-Specific Injury Is Not Related to Differences in Immune Cell Compositions Upon Poly(I:C) Treatment

**Figure 4:**
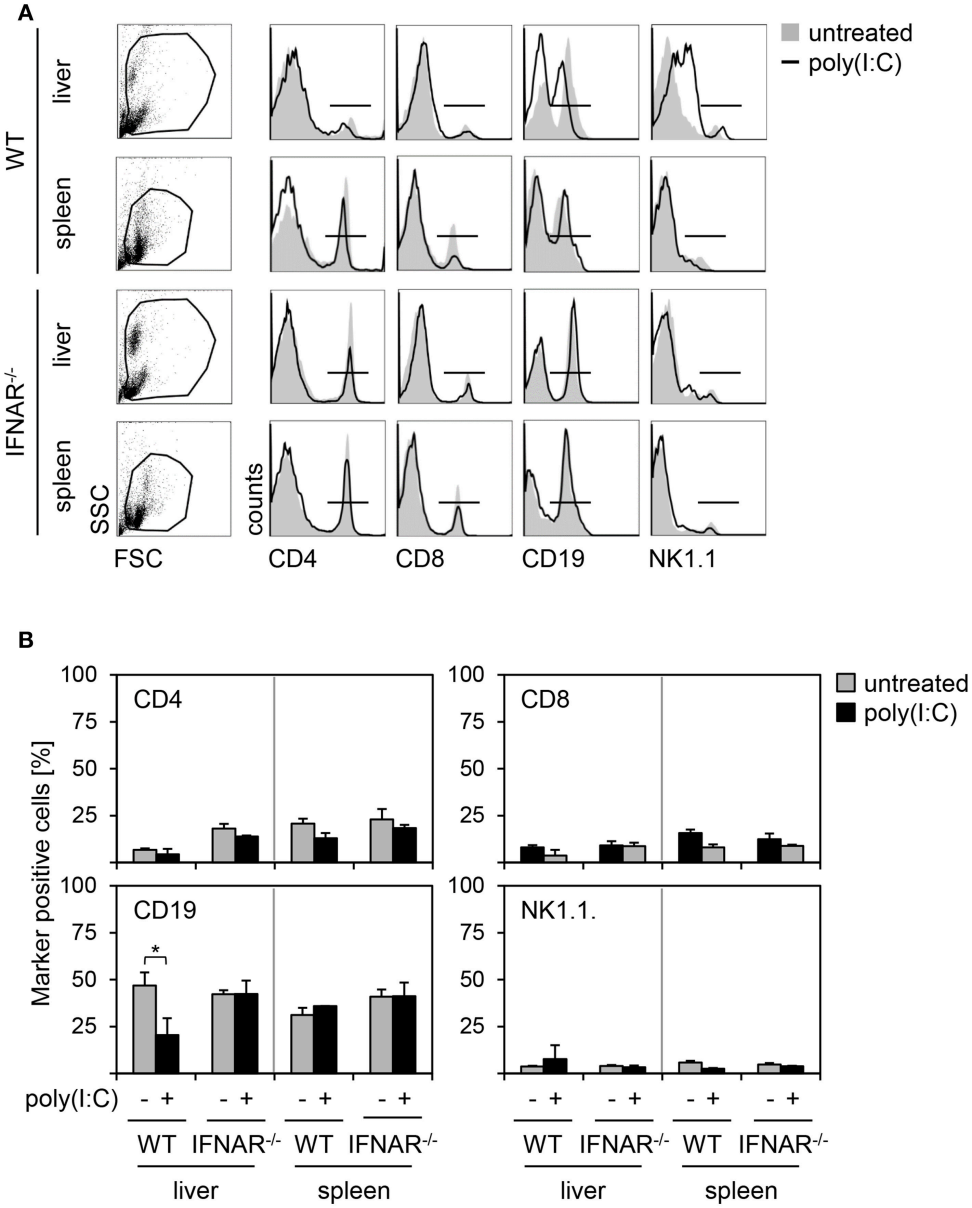
Organ-specific injury is not related to differences in immune cell compositions upon poly(I:C) treatment. **(A)** WT and IFNAR^−/−^ mice were injected with 15 μg/g BW poly(I:C). Liver perfusates and splenocytes were isolated 18 h post poly(I:C) injection and analyzed via flow cytometry. Representative dot plots of FSC/SSC are shown from untreated mice. Representative histograms for CD4^+^, CD8^+^, CD19^+^, and NK1.1^+^ cells gated on the life gate of poly(I:C)-treated mice are shown (solid lines). Untreated mice served as controls (gray shaded curves). **(B)** Proportions of CD4^+^, CD8^+^, CD19^+^, and NK1.1^+^ cells of poly(I:C)-treated mice (black bars) and untreated mice (gray bars) are given (*n* = 1–3). Error bars indicate standard deviations, ^*^ ≤ 0.05 (unpaired two-tailed *t*-test).

Next, we investigated whether different cell compositions may contribute to organ specific injury. Therefore, we analyzed the proportion of CD4^+^ T helper cells, CD8^+^ cytotoxic T cells, CD19^+^ B cells, and NK1.1^+^ natural killer (NK) cells in liver perfusates and spleen suspensions of poly(I:C)-treated WT and IFNAR^−/−^ mice. As given in Figures 4A,B, spleens of poly(I:C)-treated animals showed no differences in percentage of CD4-, CD8-, CD19-, and NK1.1-positive cells when compared to untreated controls. In addition, no differences in cell composition were observed between WT and IFNAR^−/−^ mice. Furthermore, poly(I:C) treatment had no influence on percentages of CD4-, CD8-, and NK1.1-positive cells in livers of WT and IFNAR^−/−^ mice. However, WT livers showed a significant decrease of CD19-positive cells upon poly(I:C) treatment. As described before (18), poly(I:C) treatment induces an type I IFN-dependent infiltration of MDSCs in WT but not IFNAR^−/−^ livers that influences the percentages of other cell populations. Hence, the decrease of CD19-positive cells in WT livers upon poly(I:C) treatment is most probably related to this fact. Collectively, the liver-specific injury upon poly(I:C) treatment of IFNAR^−/−^ mice is not related to differences in immune cell compositions.

### Organ-Specific Expression of IL1R1 Upon Poly(I:C) Treatment Mediates Liver Injury

**Figure 5:**
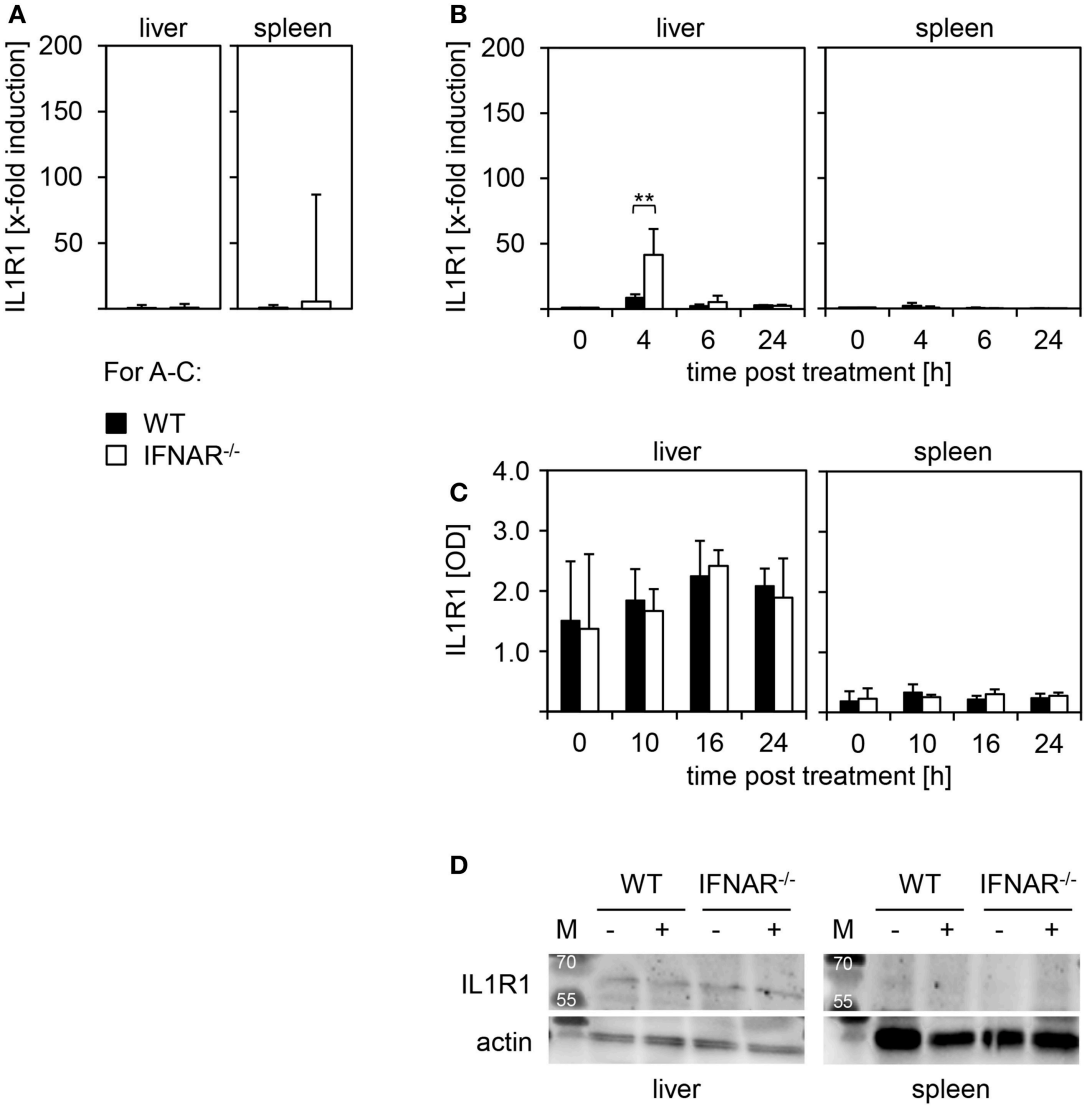
IL1R1 is expressed in livers but not spleens of WT and IFNAR^−/−^ mice. **(A)** Livers and spleens of untreated WT and IFNAR^−/−^ mice were harvested, homogenated, and tested for basal expression of IL-1R1by quantitative qRT-PCR as described in the Material and Methods section (*n* = 4–5). **(B)** Organs of poly(I:C)-treated WT and IFNAR^−/−^ mice were harvested, homogenated, and tested for fold-induction of IL1R1 expression at the indicated time points as described in the Material and Methods section (untreated *n* = 2; poly(I:C)-treated *n* = 3–5). Representative data are shown for at least three independent experiments. Error bars indicate standard deviations, ^**^ ≤ 0.01 (unpaired two-tailed *t*-test). **(C)** WT and IFNAR^−/−^ mice were treated for 10, 16, and 24 h with poly(I:C). Untreated animals served as controls. Livers and spleens were harvested, lysates were generated and tested for IL1R1 expression by ELISA (*n* = 3). **(D)** Liver and spleen lysates of untreated and poly(I:C)-treated WT and IFNAR^−/−^ mice were analyzed for IL1R1 expression by Western blot. Actin expression was analyzed as control.

To unravel the mechanism of liver-specific injury upon poly(I:C) treatment of IFNAR^−/−^ mice we investigated the level of IL1R1 expression in liver and spleen of WT and IFNAR^−/−^ mice. To ensure that basal expression of IL1R1 is comparable between genotypes, spleen and liver of untreated WT and IFNAR^−/−^ mice were harvested and mRNA was isolated. qRT-PCR analyses revealed comparable basal expression levels of IL1R1(Figure 5A). Next, WT and IFNAR^−/−^ mice were injected with poly(I:C) and fold induction of IL1R1 expression was analyzed compared to untreated control organs. For the liver and spleen of WT mice, IL1R1 expression was not increased upon treatment. However, livers of 4 h poly(I:C)-treated IFNAR^−/−^ mice showed a significant upregulation of IL1R1 expression that was not observed in IFNAR^−/−^ spleens (Figure 5B). Next, organs lysates of poly(I:C)-treated WT and IFNAR^−/−^ mice were analyzed for IL1R1 expression by an ELISA method at the indicated time points. Untreated mice served as controls. As given in Figure 5C IL1R1 expression was detected in livers of untreated and poly(I:C)-treated WT and IFNAR^−/−^ mice whereas no IL1R1 was detected in spleens. In line with this, Western blot analyses of organ lysates confirmed that IL1R1 expression was restricted to the liver (Figure 5D).

**Figure 6:**
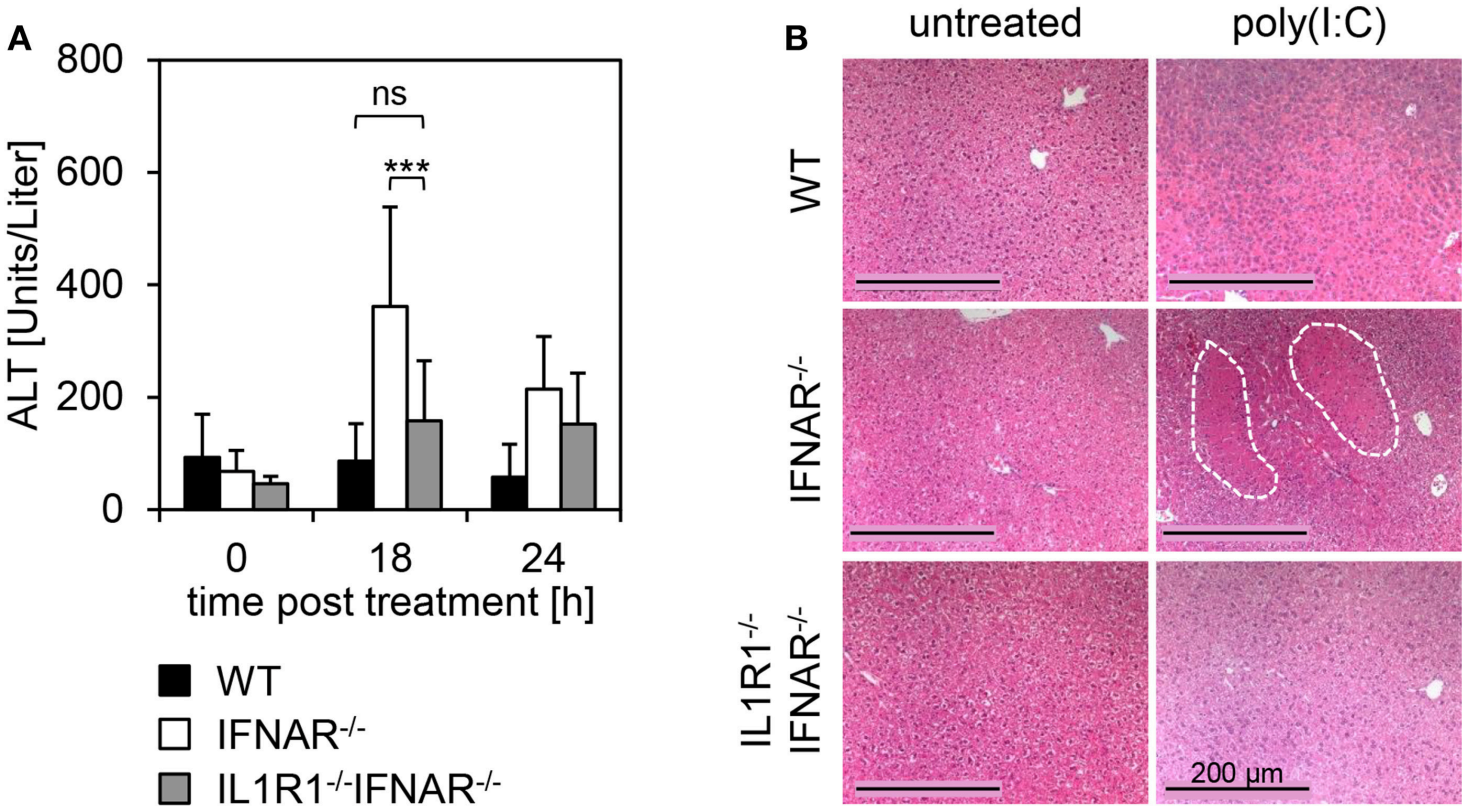
Organ-specific expression of IL1R1 mediates liver injury upon poly(I:C) treatment in IFNAR^−/−^ mice. **(A)** WT, IFNAR^−/−^, and IL1R1^−/−^IFNAR^−/−^ mice were injected with 15 μg/g BW poly(I:C). Serum was collected before injection (0 h), and 18 and 24 h post injection and ALT activity was measured. Error bars indicate standard deviations, ns, not significant; ^***^ ≤ 0.001 (unpaired two-tailed *t*-test). **(B)** Livers of untreated and poly(I:C)-treated WT, IFNAR^−/−^, and IL1R1^−/−^IFNAR^−/−^ mice were harvested. After 24 h of incubation in 10% formalin, organs were embedded in paraffin, and 2 μm slices were prepared and stained with H&E. Size standard is 200 μm. Representative sections for 3–6 mice per genotype are shown. The dotted line indicates necrotic areas.

In order to analyze whether the expression of IL1R1 in the liver is causative for the development of liver injury upon poly(I:C) treatment, mice deficient for IL1R1 were intercrossed with IFNAR^−/−^ mice to obtain IL1R1^−/−^IFNAR^−/−^ double-deficient mice. WT, IFNAR^−/−^, and IL1R1^−/−^IFNAR^−/−^ mice were injected with poly(I:C) and blood was collected at the indicated time points (Figure 6A). In stark contrast to IFNAR^−/−^ mice, IL1R1^−/−^IFNAR^−/−^ mice showed ALT activity comparable to that in WT mice 18 h post treatment. In line with this, H&E staining of organ sections of poly(I:C)-treated WT, IFNAR^−/−^, and IL1R1^−/−^IFNAR^−/−^ mice were performed. Untreated animals served as controls. As given in Figure 6B, IL1R1^−/−^IFNAR^−/−^ mice did not show any signs of liver injury and liver sections were comparable to those of WT mice. Consequently, deficiency in IL1R1 was sufficient to protect IFNAR^−/−^ mice from poly(I:C)-induced IL-1β-mediated liver injury.

In conclusion, these data suggest that organ specificity is mediated via distinct expression profiles of IL1R1. Despite high levels of harmful IL-1β in livers and spleens of IFNAR^−/−^ mice, IL1R1 is specifically expressed in the liver but not the spleen, enables binding of IL-1β to IL1R1, and finally, mediates severe liver injury.

## Discussion

Upon treatment with poly(I:C), IFNAR^−/−^ mice develop a severe liver injury while all other organs analyzed are unaffected. Liver injury is mediated by the expression of IL-1β in IFNAR^−/−^ mice that was not observed in the WT mice. In line with this, treatment with rIL-1RA rescues IFNAR^−/−^ mice from IL-1β-mediated liver injury [(18) and Figure 1]. Nevertheless, expression of high levels of IL-1β in the spleen of IFNAR^−/−^ mice did not result in injury of this organ (see Figures 1B,C). Consequently, by *in vitro* treatment of primary splenocytes and hepatocytes with poly(I:C), we excluded a direct toxic effect of poly(I:C) on hepatocytes isolated from both WT and IFNAR^−/−^ mice (Figure 2). However, *in vivo* studies showed that poly(I:C) leads to liver damage by the induction of apoptosis in hepatocytes (36, 37). Here, injection of poly(I:C) along with halothane in BALB/c mice induced apoptosis via binding on TLR3 expressed on hepatocytes (36).

It is well-accepted that besides IL-1β and IL-1RA several other cytokines are associated with liver injury as well. TGF-β participates in all stages of liver disease progression such as initial liver injury, inflammation, and fibrosis. Furthermore, studies demonstrated that TGF-β contributes to induction of apoptosis in hepatocytes as TGF-β-treated rat liver cells or human liver cell lines undergo apoptosis upon *in vitro* stimulation (26, 27, 38). IL-6 is known for its contribution to liver regeneration and is described to protect against liver damage (39). For example, IL-6 was shown to reduce acetaminophen (APAP)-induced liver injury in mice (28–30). Upon viral infection such as with MCMV, depletion of IL-10 leads to enhanced ALT activity. Here, IL-10 is associated with protection against liver damage via down-modulation of the pro-inflammatory cytokines IFN-γ and TNF-α (31, 32). Primary hepatocytes were shown to express pro-inflammatory cytokines in response to IL-17 stimulation (33). The role of IL-22 in liver injury is controversially discussed (40). In a model of APAP-induced liver damage, IL-22 was shown to increase liver damage, whereas for alcohol-induced liver injury, IL-22 ameliorated liver damage (34, 35). Nevertheless, our data indicated that expression of IFN-γ, TGF-β, IL-6, IL-10, IL-17, and IL-22 is not altered in the livers and spleens of poly(I:C)-treated WT and IFNAR^−/−^ mice (Figure 3). Hence, these cytokines are most likely not involved in poly(I:C)-induced liver injury and therefore, do not contribute to an organ-specific damage as observed in IFNAR^−/−^ mice.

The liver harbors several immune effector cells such as NK cells, T cells, and B cells that might contribute to the development of organ-specific injury in the absence of type I IFN. Several studies showed a destructive role of NK cells in the context of liver injury. As an example, treatment with low dosages of poly(I:C) induced activation and accumulation of NK cells in the liver of WT mice (41). In line with this, Ochi et al. showed that NK cells isolated from mice treated with low dosages of poly(I:C) showed strong cytotoxicity against primary hepatocytes *in vitro* (42). T cells represent, with 35%, the predominant subset of hepatic lymphocytes (43). As such, T cells are described to play a role in the onset of liver injury. For example, upon infection with Epstein-Barr virus (EBV), liver injury is induced by activated cytotoxic T lymphocytes directed against EBV-infected B cells within the liver (44). Furthermore, CD4 T cells were shown to be critical for the pro-inflammatory immune response in a model of ischemia and reperfusion-induced liver injury (45). Novobrantseva et al. showed that in the presence of B cells, CCl_4_-induced liver fibrosis developed to a much greater extent indicating that B cells participate in liver disease progression (46).

Nevertheless, the here shown analyses of the immune cell composition within spleen and liver of untreated and poly(I:C)-treated WT and IFNAR^−/−^ mice revealed no differences in percentages of NK cells, CD4^+^ and CD8^+^ T cells under all conditions tested (Figure 4). The decrease of CD19-positive cells observed in WT livers upon poly(I:C)-treatment is most likely related to the already described recruitment of MDSC to the liver of WT animals upon poly(I:C) treatment (18). CD19-positive cells are the dominant population of immune cells making up almost 50% of immune cells within liver perfusates (see Figure 4B) and therefore, it is likely that differences in CD19-positive cells upon poly(I:C) treatment are most pronounced there.

Analysis of IL1R1 revealed that expression was restricted to the liver and was not observed in spleens of WT and IFNAR^−/−^ mice (see Figures 5C,D). Hence, organ specific liver damage upon treatment with poly(I:C) is clearly determined by presence or absence of the receptor binding IL-1β. Of note, there was a slight upregulation of IL1R1 RNA expression in livers but not in spleens of IFNAR^−/−^ mice upon poly(I:C) treatment (see Figure 5B). However, this upregulation was detected on mRNA level only.

Experiments employing IL1R1^−/−^IFNAR^−/−^ double-deficient mice indicated that IL1R1 expression is indeed causative for the poly(I:C)-induced, IL-1β-mediated liver injury (Figures 6A,B). In line with this, Gehrke et al. showed that mice with a liver-specific deletion of IL1R1 exhibit reduced levels of pro-inflammatory IL-1α, IL-1β, IL-6, IFN-γ, and CCL2 in response to D-GalN/LPS treatment. Furthermore, in the absence of hepatic IL1R1, the formation of the NLRP3 inflammasome and the activation of caspase 1 were suppressed (47). This is of particular interest, since cleavage of pro-IL-1β to its bioactive form IL-1β is mediated by inflammasome-activated caspase 1 (48).

Along this line, Zhang et al. showed that in a model of APAP-induced liver injury, release of PAMPs by dying hepatocytes triggered both local and systemic inflammation involving IL1R1 signaling (49). These data indicate that organ-specific expression of IL1R1 plays an important role in the generation of liver injury. In our model, the cell type within the liver expressing IL1R1 is not yet defined and will be part of future studies.

In addition to the tissue-specific expression of the signaling IL1R1 in the liver, we observed upregulation of the decoy receptor IL1R2 in both WT and IFNAR^−/−^ spleens upon poly(I:C) treatment on mRNA level (Supplementary Figure 1). Furthermore, IL-1RA protein expression was enhanced upon poly(I:C) treatment in WT livers (18) and both WT and IFNAR^−/−^ spleens but not IFNAR^−/−^ livers (data not shown). Even though we showed that IL1R1 deficiency is sufficient to protect IFNAR^−/−^ mice from poly(I:C)-induced liver damage (see Figure 6), the expression of IL-1RA and IL1R2 in protected organs or genotypes, respectively, might also contribute to protection. Collectively, type I IFNs regulate several members of the IL-1family, preventing the IL-1-mediated damage in liver and spleen. In absence of an intact type I IFN signaling, the contact of an organism with PAMPs such as double-stranded RNA can lead to an imbalanced expression of IL-1family members, causing severe, organ-specific tissue damage.

In conclusion, innate immune responses within the liver are known to play an important role in host defense against invading microorganisms. Nevertheless, dysregulation of these organ-specific immune responses leads to massive liver damage. Insights in the local immune mechanisms of the liver, particularly the liver-specific expression of IL1R1, will help to develop novel treatment options for patients with severe liver injury.

## Ethics Statement

This study was carried out in accordance with the recommendations of German animal welfare. The protocol was approved by the Regierungspräsidium Darmstadt.

## Author Contributions

MA designed experiments, performed experiments, analyzed data, and wrote the paper. TR and ES designed experiments, performed experiments, and analyzed data. SK and CD performed experiments and analyzed data. MN, EK, MW, SS, and SW performed experiments. DK cross-bred mice. MB designed experiments. ZW designed experiments, analyzed data, and wrote the paper.

### Conflict of Interest Statement

The authors declare that the research was conducted in the absence of any commercial or financial relationships that could be construed as a potential conflict of interest.

## Abbreviations

ALT: alanine aminotransferase
BW: body weight
H&E: hematoxylin and eosin
hpi: hours post injection
IFN: interferon
IFNAR: type I IFN receptor
IL: interleukin
IL1R1: IL-1 receptor type 1
IL1R2: IL-1 receptor type 2
IL-1RA: interleukin-1 receptor antagonist
i.p.: intraperitoneal
ISG: IFN-stimulated genes
MDSC: myeloid-derived suppressor cell
NK: natural killer
PBS: phosphate-buffered saline
r: recombinant
RIG-I: retinoic acid-inducible gene 1
RLR: RIG-like receptors
TGF-β: transforming growth factor beta
TLR: Toll-like receptor
WT: wild type.
