# Supplementary figures and images for: Organ-Specific Expression of IL-1 Receptor Results in Severe Liver Injury in Type I Interferon Receptor Deficient Mice
Source: Front Immunol. 2019 May 9;10:1009. doi: 10.3389/fimmu.2019.01009 (PMC6521796; doi:10.3389/fimmu.2019.01009)

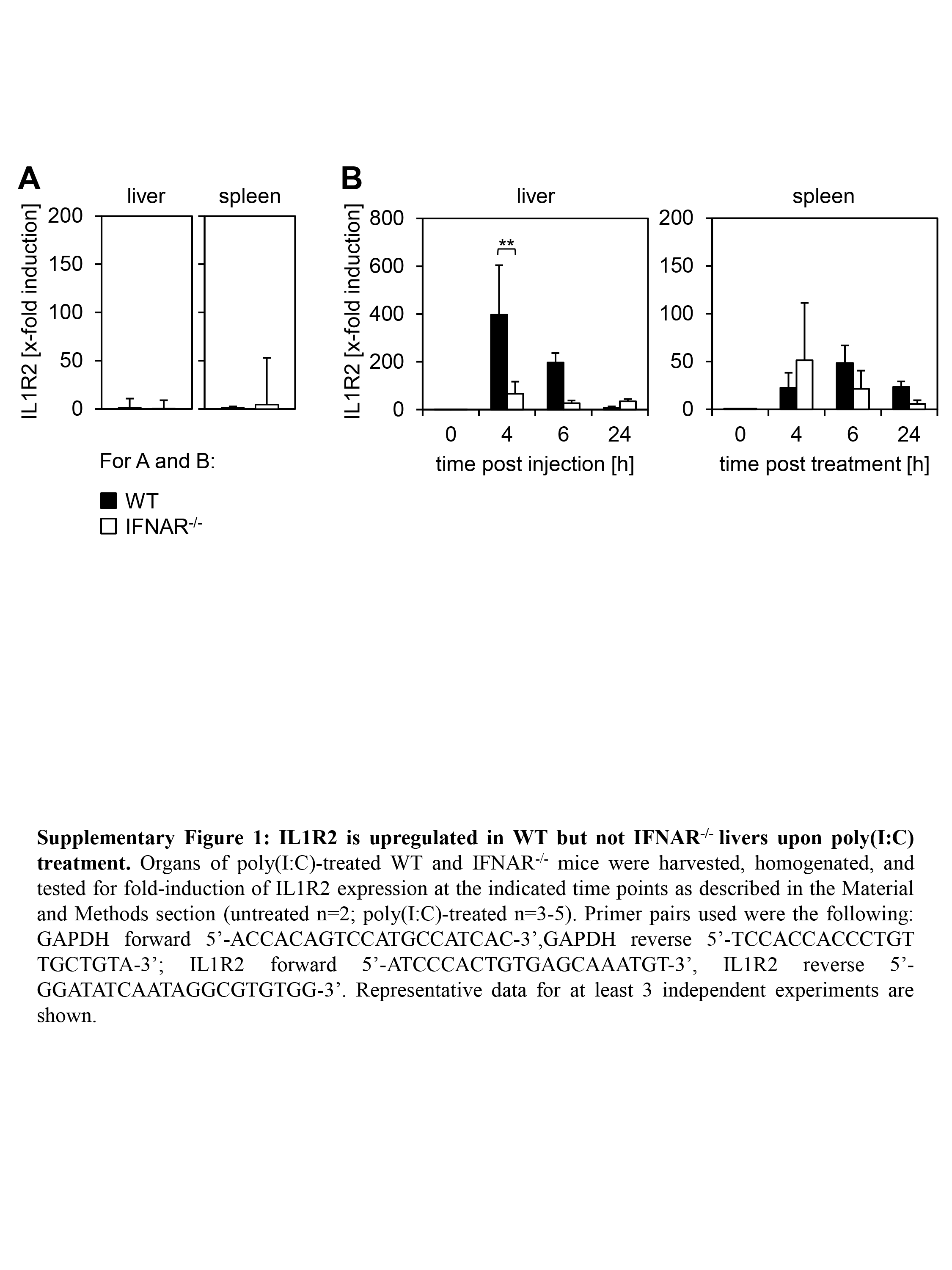

Supplement: Supplementary file 1 [file Image_1.TIF]
